# Patterns of Social, Economic, And Health Challenges Due to the COVID-19 Pandemic Experienced by Latino Communities: A Latent Class Analysis

**DOI:** 10.1007/s40615-025-02514-6

**Published:** 2025-06-27

**Authors:** Gabriel Ramirez, Yinzhou Ou, Ronald Saxton, Alejandra F. Miller, Melissa M. Cuesta, Ana O. Meza, Suzanne Grieb, Kathleen R. Page, Cui Yang

**Affiliations:** 1 Department of Medicine, Johns Hopkins School of Medicine, Baltimore, MD USA; 2 Department of Pediatrics, Johns Hopkins University School of Medicine, Baltimore, MD USA; 3 Department of Health Behavior, Society and Policy, Rutgers School of Public Health, Piscataway, NJ USA

**Keywords:** Latino, COVID-19, Mental health, Latent class analysis, Health inequity

## Abstract

**Objective:**

Latino communities are diverse and necessitate in-depth knowledge of the heterogeneity of Latinos beyond a singular race or ethnicity. Each subgroup may have been impacted by the COVID-19 pandemic differently. This study examined the patterns of challenges that Latinos faced due to the COVID-19 pandemic, identified subgroups with diverse needs, and explored the association between these subgroups and health outcomes.

**Methods:**

A total of 720 participants enrolled between August 2022 and August 2023 as part of the Rapid Acceleration of Diagnostics—Underserved Populations (RADx-UP) initiative in Maryland. Latent class analysis (LCA) was employed to identify subgroups of participants based on self-reported indicators of challenges related to the COVID-19 pandemic, including access to health care, housing, food security, access to clean water, medication, and transportation. Health outcomes included self-report health, anxiety, and depression screening. Logistic regressions were conducted to examine the relationships between class membership, sociodemographic characteristics, and various health outcomes.

**Results:**

A four-class latent class model was identified: *Class 1* (31.67%) reported no challenge across all indicators; *Class 2* (32.62%) reported minor challenges in accessing health care and medicine and food security; *Class 3* (35.72%) reported minor challenge in all indicators, and *Class 4* (22.28%) reported major challenges across all indicators. Not having health insurance, fewer years of living in the US, and not being proficient in English were associated with belonging to a group that faced increased challenges due to the COVID-19 pandemic. Class memberships were associated with health outcomes, including depression and anxiety.

**Conclusions:**

This study found that pandemic-associated challenges were highly heterogeneous among Latinos, which were variably associated with health outcomes, including depression and anxiety. Findings from the current research can inform post-pandemic resource allocation and future pandemic preparedness to address longstanding structural inequities. Continued investments are needed to address long-lasting social and economic hardship and public health issues, such as mental health, among the most vulnerable communities.

## Introduction

As of 2022, the United States (US) is home to roughly 63 million Latinos, constituting the largest racial or ethnic minority group in the country and accounting for 19.1% of the total population [1]. The COVID-19 pandemic revealed abiding inequalities in the US [2]. The Latino communities were disproportionately impacted by higher COVID-19 prevalence, COVID-19 related mortality [3], hospitalization, and intensive care unit admission [4, 5]. Mental health among Latinos also emerged as a significant public health concern during the pandemic, with national surveys indicating elevated levels of anxiety, depression, and suicidal ideation [6]. The COVID-19 pandemic also resulted in economic and social impacts on Latino communities. In 2022, 75% of Latinos reported difficulty with household expenses, and they were approximately twice as likely to report food and housing payment insecurity as Whites [7]. Those economic, social, and health hardships are likely to persist after the COVID-19 public health emergency ended in May 2023.

Despite being commonly viewed as a single ethnic category, Latino communities are diverse and necessitate in-depth knowledge of the heterogeneity of Latino identity beyond a singular race or ethnicity [8]. In addition to differences in country of origin or spoken language, Latino subgroups exhibit significant variability in socioeconomic status (SES), such as educational attainment, income levels, geographic distribution, and immigration status [9]. As a result, each subgroup may have been impacted by the COVID-19 pandemic differently. During the COVID-19 pandemic, the US government enacted numerous major appropriations laws and provided funding to the US to respond to the nationwide health crisis and economic devastation. For example, the Coronavirus Aid, Relief, and Economic Security (CARES) Act introduced several health-related measures to support pandemic response efforts, including guaranteed coverage for COVID-19 testing and treatment, regardless of immigration or insurance status [10]. However, concerns about potential immigration-related repercussions may have discouraged many non-citizen Latinos from seeking COVID-19 health services [11].

A better understanding of the unique needs of the most vulnerable subgroups can inform the allocation of limited resources for essential social, economic, and public health services. To the best of our knowledge, no published data has documented the heterogeneity of social, economic, and health hardships due to the COVID-19 pandemic in Latino communities. The current study employed latent class analysis (LCA) to identify subgroups of Latinos based on the varying levels of challenges they faced due to the COVID-19 pandemic. LCA performs considerably better than other clustering methods because it identifies sub-groups within a population based on sentinel patterns of co-occurrence among characteristics hypothesized as indicators of a latent construct. In contrast to investigator-determined categories, LCA involves a series of diagnostic criteria that help to determine the appropriate number of groups within a study sample [12]. In this respect, LCA enables us to identify patterns of hardship related to COVID-19 pandemic indicators without imposing our own criteria on the data, thereby ensuring a more accurate representation of the experiences of Latino communities.

The objectives of this study were to (1) characterize various patterns of challenges due to the COVID-19 pandemic among Latino communities and (2) identify how heterogeneous experiences are associated with health-related outcomes. The overall goals of this study were to understand COVID-19-related challenges experienced by different subgroups within Latino communities and their impact on health outcomes and to inform post-pandemic resource allocation and policies for future pandemic preparedness.

## Methods

### Study Design and Data Collection

Participants for the current study were recruited and enrolled via peer referral and social marketing between August 2022 and August 2023 as part of the RADx® Underserved Populations (RADx-UP) initiative. The National Institutes of Health (NIH) launched the RADx-UP program to enhance understanding of strategies for improving access to COVID-19 testing, with a focus on communities most severely affected by the pandemic [13]. Eligible participants in our study were self-identified as Latino or Hispanic, Maryland residents, and at least 18 years old. After providing oral consent over the phone or in person, each participant completed a survey instrument either over the phone or in person. The survey was offered in English or Spanish, based on the participant’s preference. Participants received $30 for completing the survey. All study protocols were approved by the Institutional Review Board before implementation.

### Measures

The survey included indicators of COVID-19-related challenges participants experienced in the past 6 months, health-related outcomes, and sociodemographic characteristics. The COVID-19-related challenges were part of NIH RADx-UP Common Data Elements (CDE) [14]. They were assessed by asking participants, “The COVID-19 pandemic may cause challenges for some people, whether they got COVID-19 or not. In the past six months, have you or your family experienced any of the challenges below?” The challenges indicators included six indicators: (1) access to health care (“Getting the health care I need, including for mental health”), (2) housing (“Having a place to stay/live”), (3) food security (“Getting enough food to eat”), (4) access to clean water (“Having clean water to drink”), (5) medication access (“Getting the medicine I need”), and (6) access to transportation (“Getting to where I need to go”). The response options included “no, not a challenge,” “yes, a minor challenge,” and “yes, this is a major challenge.”

Health-related outcomes included self-reported health (excellent, very good, good, fair, and poor), the Generalized Anxiety Disorder 2-item (GAD-2) for anxiety screening [15], and the Patient Health Questionnaire-2 (PHQ-2) for depression screening [16]. Other measures included vaccination status for the current seasonal influenza (Flu) and COVID-19, as well as having ever taken a COVID-19 test.

Sociodemographic characteristics included age, sex at birth, employment status, housing situation, foreign-born status, internet access at home, health insurance coverage, and years of residency in the US. English proficiency was assessed by asking participants, “How well do you speak English?” with response options “very well,” “well,” “not well,” and “don’t speak English.”

### Statistical Analysis

Descriptive statistics were used to describe participants’ sociodemographic characteristics and health-related outcomes. Latent class analysis (LCA) using STATA was employed to identify subgroups of participants based on the challenges they experienced due to the COVID-19 pandemic. To determine the number of latent classes that best represented the data, we compared models with different class solutions. We examined goodness-of-fit statistics for each model, including the Akaike information criterion (AIC), Bayesian information criterion (BIC), the likelihood ratio chi-square, and entropy. Lower values of AIC and BIC indicate a better fit of the model to the data, while a non-significant *p*-value for the chi-square statistic suggests that the model fits the data as well as a saturated model. Higher entropy denotes better class separation.

Following the identification of the latent classes, we proceeded with univariate and multivariate regression to explore the associations between class membership, sociodemographic characteristics, and different health outcomes. This approach enabled us to estimate the odds ratios (OR) and 95% confidence intervals (95% CI) for class membership relative to the reference class (i.e., the least challenged class). The multivariate regression models included covariates with a *p*-value less than 0.10 in univariate analysis. We attempted to develop a parsimonious multivariate model that included all key variables. Therefore, we took a less strict approach by including variables with a *p*-value less than 0.10 rather than 0.05.

## Results

### Participant Characteristics

Table 1 illustrates a summary of the participant characteristics. Among the 720 participants, 73.8% were female, with an average age of 40.6 years, ranging from 18 to 82 years. Over half of the participants had an educational attainment lower than a college degree (51.9%). Although over two-thirds (69.0%) were employed, 60.3% did not have health insurance. The majority (94.7%) were foreign-born, with an average of 15 years spent living in the US, and over half (54.0%) were not proficient in English. Despite the majority of the sample self-reporting their health status as excellent (5.3%), very good (7.8%), or good (44.3%), over a fifth (22.0%) screened positive for anxiety and 17.2% for depression. The proportion of individuals who had ever taken a COVID-19 test (78.8%) and received a vaccination (87.4%) among the sample was high, and 42.5% had received the current seasonal flu vaccine.

**Table 1:** Participant characteristics(*n*** = **720)

*N* = 720	Frequency	Percent
Age: mean (range)	40.67 (18, 82)	
Sex		
Male	189	26.2%
Female	531	73.8%
Education		
Lower (< college)	374	51.9%
College or above	346	48.1%
Employment status		
Not employed	223	31.0%
Employed	497	69.0%
Housing		
Rent or sublet a room, stay in a shelter or live in street	84	11.7%
Own or rent own house or apartment	636	88.3%
Internet access at home		
No	92	12.8%
Yes	628	87.2%
Health insurance		
No	434	60.3%
Yes	286	39.7%
Foreign born		
No	38	5.3%
Yes	682	94.7%
# of years living in the US*: mean (range)	15.04 (0, 74)	
English proficiency		
Not proficient	389	54.0%
Proficient	331	46.0%
Self-reported health		
Excellent	38	5.3%
Very good	56	7.8%
Good	319	44.3%
Fair	270	37.5%
Poor	37	5.1%
GAD-2 generalized anxiety disorder screening		
No	547	78.0%
Yes	173	22.0%
PHQ-2 depression screening		
No	596	82.8%
Yes	124	17.2%
Ever taken COVID-19 test		
No	153	21.3%
Yes	567	78.7%
Had COVID-19 vaccine		
No	91	12.6%
Yes	629	87.4%
Had current seasonal flu vaccine		
No	414	57.5%
Yes	306	42.5%

^*^For those born in the US, using self-reported age

### Latent Class Analysis Results

Upon examining the goodness-of-fit statistics, a 4-class model was determined to have a better fit due to its lower AIC and BIC values, despite the Chi-Square statistic suggesting an adequate fit for both models and the elevated entropy indicating better class separation. Table 2 illustrates model fit statistics for 1-, 2-, 3-, and 4-class models. Following a discussion among the study team, which included community health workers with extensive experience in Latino communities, a 4-class model was selected for further analysis. This 4-class model provided a more nuanced segmentation of the challenges, further supported by the proportion of participants in each class and the probability of reporting challenges across various indicators (Table 3).

**Table 2:** Model fit information for latent class analysis models

	1-Class model	2-Class model	3-Class model	4-Class model
Likelihood ratio chi-square^*^	2,012.29	1050.36	644.92	551.90
*P* value for the chi-squared statistic^**^	< 0.01	< 0.01	0.89	1.00
AIC	8390.99	7455.06	7075.62	7008.60
BIC	8445.94	7569.54	7249.63	7242.14
Entropy^***^	N/A	0.786	0.806	0.801

^*^A lower chi-squared value generally indicates a better fit. However, it is relative and should be interpreted in the context of the degrees of freedom and the size of your dataset

^**^A high *P* value (usually > 0.05) suggests that there is no significant difference between your model and the saturated model, indicating a good fit

^***^Higher entropy denotes better class separation

**Table 3:** Four class model—COVID-19-related challenge classes

Challenge/area	Class 1: no challenge in all indicators	Class 2: minor challenge in healthcare, food security and medicine	Class 3: minor challenge in all indicators	Class 4: major challenge in all indicators
Access to health care				
Not a challenge	0.85	0.34	0.17	0.18
Minor challenge	0.08	0.42	0.63	0.20
Major challenge	0.07	0.23	0.20	0.61
Housing				
Not a challenge	0.92	0.87	0.23	0.28
Minor challenge	0.05	0.09	0.63	0.14
Major challenge	0.03	0.04	0.14	0.58
Food security				
Not a challenge	0.92	0.34	0.05	0.04
Minor challenge	0.07	0.55	0.85	0.11
Major challenge	0.01	0.11	0.10	0.84
Access to clean water				
Not a challenge	0.97	0.92	0.26	0.42
Minor challenge	0.02	0.06	0.71	0.12
Major challenge	0.01	0.01	0.03	0.46
Access to medicine				
Not a challenge	0.98	0.22	0.16	0.08
Minor challenge	0.02	0.59	0.73	0.08
Major challenge	0	0.19	0.11	0.84
Access to transportation				
Not a challenge	0.92	0.62	0.29	0.20
Minor challenge	0.05	0.20	0.54	0.08
Major challenge	0.03	0.18	0.16	0.72
Class size	31.67%	32.62%	13.44%	22.27%

Table 3 describes the patterns of COVID-19-related challenges in each class identified by the 4-class model. Based on how participants reported the levels of challenges in each indicator, “not a challenge,” “minor challenge,” or “major challenge,” we categorized them into four groups: (1) least challenged in all indicators; (2) minor challenged in some indicators, such as access to healthcare, food security, and access to medications; (3) minor challenged in all indicators; and (4) major challenged in all areas.

More specifically, *Class 1- no challenges in all indicators* (31.67% of the sample) stood out as the group with a high probability of responding “not a challenge” across all indicators. *Class 2- minor challenges in some indicators* (32.62% of the sample) was characterized by a high probability of reporting “a minor challenge” in certain indicators, particularly in accessing health care (0.42), food security (0.55), and obtaining medicine (0.59). Meanwhile, this class also maintained a relatively high probability of reporting “no challenge” in having a place to live (0.87), accessing clean water (0.92), and accessing transportation (0.62). *Class 3-minor challenge in all indicators* (13.44% of the sample) was marked by a high probability of reporting “a minor challenge” across all indicators. Finally, *Class 4- major challenge in all indicators* (22.28% of the sample) depicted the group most impacted by COVID-19, with a high probability of reporting major challenges in all indicators. A considerably high probability of encountering major challenges in access to health care (0.61), food security (0.84), stable housing (0.58), access to medicine (0.84), and access to transportation (0.72).

### Association Between Sociodemographic Characteristics and LCA Groups

The relationships between various sociodemographic factors and class membership are presented in Table 4. Using *Class 1—no challenges in all indicators* as a reference group, the multinomial regression model revealed that younger age, female sex, lower education attainment, unemployed, less stable housing, no access to internet at home, not having health insurance, fewer years of living in the US, and not being proficient in English were all significantly associated with belonging to the *Class 4—major challenge in all indicators*. In addition, not having health insurance, fewer years of living in the US, and not being proficient in English were associated with belonging to *Class 2 (minor challenges in some indicators)* or *Class 3 (minor challenge in all indicators)*.

**Table 4:** Multinomial logistic regression of 4-Class membership (using Class 1: no challenge as a reference group)

	Class 2: minorchallenges in someareas		Class 3: minorchallenges in allindicators		Class 4: majorchallenges in allindicators	
	Coefficient(95% CI)	*p*-value	Coefficient(95% CI)	*p*-value	Coefficient(95% CI)	*p*-value
Age	0.03 (−0.04, −0.01)	0.004	−.01 (−0.03, 0.01)	0.25	−0.02 (−0.04, −.001)	0.009
Sex at birth Male Female	0.43 (−0.06, 0.93)	0.087	0.84 (0.15, 1.52)	0.017	0.81 (0.29, 1.33)	0.002
Housing Rent or sublet a room, stay in a shelter or live in street Own or rent own house or apartment	0.57 (−0.29, 1.44)	0.20	0.80 (−0.31, 1.90)	0.15	−0.84 (−0.42, −0.26)	0.005
Access to internet at home No Yes	−0.55 (−1.31, 0.22)	0.16	−0.59 (−1.46, 0.27)	0.18	−0.97 (−1.68, −0.28)	0.006
Having health insurance No Yes	−1.15 (−1.64, − 0.67)	<0.001	−1.28 (−1.84, − 0.73)	<0.001	−1.69 (− 2.18, − 1.21)	<0.001
# of years living in the US^a^	−.06 (−.08, −.04)	<0.001	−.05 (−.08, −.03)	<0.001	−0.10 (−0.12, − 0.07)	<0.001
English proficiency No Yes	−0.45 (−0.91, 0.01)	0.05	−1.14 (−1.72, −0.56)	<0.001	−0.53 (−2.03, −1.03)	< 0.001

^*^For those born in the US, using self-reported age

### Association Between Sociodemographics, Health-related Outcomes, and Class Membership

The relationship between latent class membership, sociodemographic factors, and health-related outcomes is presented in Table 5, with *Class 1 (no challenges in all indicators*) as the reference group. Multivariate models indicated that members of *Class 2 (minor challenges in some indicators)* were more likely to have screened positive for depression (AOR 2.42, 95% CI 1.4–4.21) and more likely to have a COVID-19 test (AOR 167, 95% CI 1.02–2.72). Members in *Class 3 (minor challenge in all indicators)* exhibited higher odds of self-reporting poor health (AOR 2.66, 95% CI 1.65, 4.31), being screened positive for anxiety (AOR 2.41, 95% CI 1.37, 4.24) and for depression (AOR 3.17, 95% CI 1.62, 6.21). *Class 4 (major challenge in all indicators)* only had significantly higher odds of being screened positive for depression (AOR 2.42, 95% CI 1.33, 4.41).

**Table 5:** Univariate and multivariate regression between class membership and outcomes of interest (*n* = 720)

	Self report poor health		Anxiety screening		Depression screening		Covid test		Covid vaccine		Seasonal flu	
	OR (95% CI)	AOR (95% CI)	OR (95% CI)	AOR (95% CI)	OR (95% CI)	AOR (95% CI)	OR (95% CI)	AOR (95% CI)	OR (95% CI)	AOR (95% CI)	OR (95% CI)	AOR (95% CI)
Age	1.01 (1.00 − 1.02) +	1.01 (1.00 − 1.02) *	1.00 (0.99 − 1.02)	–	1.00 (0.98 − 1.01)	–	1.02 (1.01 − 1.04) **	1.02 (1.00 − 1.04) +	1.04 (1.02 − 1.06) **	1.05 (1.02 − 1.07) **	1.03 (1.02 − 1.05) **	1.03 (1.02 − 1.05) **
Sex at birth Male Female	1.63 (1.21 − 2.25) **	1.49 (1.08 − 2.05) *	1.66 (1.09 − 2.52) *	1.44 (0.94 − 2.22) +	1.40 (0.88 − 2.22)	–	1.56 (1.06 − 2.30) *	1.46 (0.96 − 2.22) +	1.55 (0.97 − 2.47) +	1.40 (0.85 − 2.30)	1.56 (1.10 − 2.19) *	1.52 (1.06 − 2.18) *
Education High school or lower College or higher	0.59 (0.45 − 0.78) **	0.64 (0.48 − 0.85) **	0.83 (0.59 − 1.17)	–	0.80 (0.54 − 1.19)	–	1.42 (0.99 − 2.04) +	1.03 (0.69 − 1.55)	1.65 (1.05 − 2.59) *	1.59 (0.98 − 2.57) +	1.20 (0.89 − 1.61)	–
Employment Currently not working Currently working	0.84 (0.63 − 1.13)	–	0.67 (0.47 − 0.97) *	0.73 (0.50 − 1.05) +	0.49 (0.33 − 0.73) **	0.52 (0.34 − 0.77) **	1.15 (0.78 − 1.68)	–	1.18 (0.74 − 1.87)	–	0.85 (0.62 − 1.17)	–
Housing Rent or sublet a room, stay in a shelter or live in street Own or rent own house or apartment	1.68 (1.08 − 2.61) *	1.43 (0.91 − 2.24)	1.39 (0.78 − 2.47)	–	1.05 (0.57 − 1.92)	–	1.47 (0.88 − 2.46)	–	1.76 (0.97 − 3.19) +	1.79 (0.93 − 3.45) +	0.93 (0.59 − 1.47)	–
Access to internet at home No Yes	0.98 (0.65 − 1.48)	–	0.88 (0.53 − 1.45)	–	0.62 (0.37 − 1.04) +	0.73 (0.42 − 1.25)	2.81 (1.76 − 4.47) **	2.25 (1.37 − 3.68) **	2.01 (1.15 − 3.52) *	1.73 (0.95 − 3.13) +	1.92 (1.20 − 3.10) **	1.59 (0.97 − 2.62) +
Having health insurance No Yes	0.78 (0.59 − 1.04) +	0.90 (0.66 − 1.23)	1.30 (0.92 − 1.83)	–	1.03 (0.69 − 1.53)	–	3.92 (2.51 − 6.14) **	3.23 (1.92 − 5.43) **	2.27 (1.37 − 3.76) **	2.05 (1.14 − 3.70) *	2.33 (1.71 − 3.16) **	2.08 (1.46 − 2.96) **
# of years living in the US^a^	1.01 (1.00 − 1.02)	–	1.00 (0.98 − 1.01)	–	0.99 (0.97 − 1.00)	–	1.03 (1.01 − 1.05) **	0.99 (0.97 − 1.02)	1.02 (1.00 − 1.04) +	0.98 (0.95 − 1.01)	1.02 (1.01 − 1.04) **	0.99 (0.97 − 1.01)
English proficiency No Yes	0.84 (0.64 − 1.11)	–	1.05 (0.74 − 1.47)	–	0.85 (0.58 − 1.26)	–	2.44 (1.66 − 3.59) **	1.90 (1.20 − 3.01) **	1.35 (0.86 − 2.11)	–	1.10 (0.82 − 1.48)	–
Latent class												
Class 1 (no challenge)												
Class 2 (minor challenge in food, health care and medicine)	1.53 (1.09 − 2.13) *	1.39 (0.98 − 1.96) +	1.61 (1.03 − 2.51) *	1.55 (0.99 − 2.43) +	2.49 (1.44 − 4.30) **	2.42 (1.40 − 4.21) **	1.14 (0.73 − 1.78)	1.67 (1.02, 2.72)*	0.91 (0.52 − 1.60)	1.05 (0.58 − 1.91)	0.67 (0.46 − 0.96) *	0.79 (0.54 − 1.17)
Class 3 (minor challenge)	3.04 (1.90 − 4.86) **	2.66 (1.65 − 4.31) **	2.56 (1.46 − 4.48) **	2.41 (1.37 − 4.24) **	3.23 (1.66 − 6.29) **	3.17 (1.62 − 6.21) **	0.91 (0.51 − 1.64)	1.35 (0.71,2.57)	0.48 (0.25 − 0.93) *	0.52 (0.26 − 1.07) +	0.80 (0.49 − 1.32)	0.92 (0.54 − 1.57)
Class 4 (major challenge)	1.55 (1.05 − 2.30) *	1.35 (0.89 − 2.05)	1.69 (1.03 − 2.76) *	1.56 (0.94 − 2.56) +	2.66 (1.47 − 4.80) **	2.42 (1.33 − 4.41) **	0.96 (0.59 − 1.57)	1.71 (0.98,2.99) +	1.04 (0.55 − 2.00)	1.41 (0.69 − 2.90)	0.69 (0.46 − 1.05) +	0.86 (0.54 − 1.36)

^a^For those born in the US, using self-reported age

+ *p* < 0.10, *p < 0.05, **p < 0.001

Several associations between sociodemographic factors and health-related outcomes are notable in the multivariate models. Female participants demonstrated higher odds of self-reporting poor health (AOR 1.49, 95% CI 1.08–2.05) and higher odds of having received a seasonal flu shot (AOR 1.52, 95% CI 1.06–2.18) than their male counterparts. Individuals with college or higher education had lower odds of reporting poor health (AOR 0.64, 95% CI 0.48–0.85). Employed participants demonstrated lower odds of being screened positive for depression (AOR 0.52, 95% CI 0.34–0.77) than those who were unemployed. In terms of COVID-19 testing, participants with access to the internet at home (AOR 2.25, 95% CI 1.37, 3.68), those having health insurance (AOR 3.23, 95% CI 1.92, 5.43), and English proficient (AOR 1.90, 95% CI 1.20, 3.01) were more likely to have ever had COVID-19 tests. Finally, insured participants demonstrated higher odds of receiving the COVID-19 vaccine (AOR 2.05, 95% CI 1.14–3.70) and the current seasonal flu vaccine (AOR 2.08, 95% CI 1.46–2.96).

## Discussion

This study demonstrated that experiencing challenges due to the COVID-19 pandemic was highly heterogeneous among a community sample of Latinos who were predominantly foreign-born. Specifically, we identified four subgroups facing different levels of challenges, which were variably associated with health-related outcomes, including depression and anxiety. Latinos without health insurance, those with limited English proficiency, and those who are newly arrived in the US faced the most significant challenges. We conducted this study during and after the COVID-19 public health emergency. These findings contribute to the limited literature examining the heterogeneous and potentially long-term effects of the COVID-19 pandemic on social and economic stability, as well as health outcomes, among Latinos.

Using LCA, we identified four subgroups facing various challenges due to the COVID-19 pandemic. Although close to one-third of participants (31.67%) reported no challenges (*Class 1*), 32.62% of the sample (*Class 2*) experienced minor challenges in accessing health care and medicine and food security, and 35.72% (*Class 3 and Class 4*) experienced either minor challenges or major challenges across all indicators. We identified several sociodemographic differences among class membership groups. Notably, individuals without health insurance, those with fewer years of living in the US, and those who are not proficient in English are all associated with membership in a group facing elevated challenges due to COVID-19. Although we did not assess immigration status, this result corroborated similar findings from other studies [17, 18] that pandemic-related economic hardship was more profound among non-citizens, many of whom were excluded from federal and state economic relief measures.

Differences in levels of hardship are evident and, to some extent, reflect longstanding disparities that may have existed even before the pandemic. Hardship across various social, economic, and health domains during an unprecedented global pandemic indicates multiple stressors, resulting in accumulated stress among individuals who have already faced tremendous challenges before the COVID-19 pandemic. The pandemic exacerbated existing structural inequities [19]. We also found that class memberships are associated with health-related outcomes. More specifically, participants reporting minor challenges in all indicators were more likely to report poor health and to be screened positive for anxiety and depression. Surprisingly, participants reporting major challenges on all indicators only had significantly higher odds of being screened positive for depression. One explanation might be that those reporting significant challenges were more resilient or had developed positive coping mechanisms in response to the hardships during the pandemic, serving as buffers against adverse health outcomes. Further research is needed to investigate the underlying processes that contribute to resilience during the pandemic.

Several limitations should be noted. First, all participants were recruited from the state of Maryland. Therefore, our results may not be generalizable to Latinos in other parts of the US, as COVID-19-related experiences among Latino communities varied by region [20]. However, community-based recruitment strategies facilitate the inclusion of diverse subgroups within the broader Latino population. Second, self-report data might be subject to social desirability and recall biases. Third, cross-sectional data did not imply causality between class membership and health outcomes. Further investigation should explore the causal inferences of these associations and evaluate potential pathways through which hardship due to a pandemic may influence health. Fourth, we measured only six indicators of COVID-19-related challenges as part of the NIH RADx-UP common data elements. We may have missed other key indicators related to economic and health hardship. Fifth, we did not assess participants’ experience before the pandemic, which can be a critical confounder. Finally, we did not assess resilience factors [17], such as social support and family cohesion, which can be essential modifiers to mitigate the economic hardship and adverse health effects caused by the pandemic.

Despite these limitations, our findings confirm that there are subgroups in Latino communities experiencing elevated social, economic, and health hardships due to the COVID-19 pandemic. In addition, health outcomes, particularly mental health, are impacted by those social and economic factors outside the healthcare system, which may play an even more profound role in shaping health. Economic support provided by federal legislation during the COVID-19 public health emergency has mitigated some of the pandemic’s health and economic effects. Continued investments are needed to address potential long-lasting social and economic hardship and public health issues, such as mental health, and prepare for future pandemics. Reinstating exclusionary policies and practices from the pre-pandemic period may undermine the trust established with marginalized communities and jeopardize the effectiveness of future public health interventions [19]. Broader legislative initiatives should expand economic support and health coverage to all individuals, regardless of their immigration status. The path to health equity will not be achieved unless we start addressing its root causes with preventive measures and policies that address longstanding structural inequities.

## Data Availability

The datasets and materials in the current study are available from the corresponding author upon reasonable request
